# Gaining Soft Tissue with a Hydrogel Soft Tissue Expander: A Case Report

**DOI:** 10.1055/s-0042-1749156

**Published:** 2022-10-04

**Authors:** Henri J.J. Uijlenbroek, Yuelian Liu, Daniel Wismeijer

**Affiliations:** 1 Department of Oral Cell Biology, Academic Centre for Dentistry Amsterdam, Amsterdam, The Netherlands; 2 Private Practice, The Netherlands

**Keywords:** hydrogel, tissue expander, amalgam tattooing, bone augmentation, implant

## Abstract

In this case report, we describe the treatment of a patient referred to our clinic with a hopeless tooth 21 with an attached pontic. The aim of this case report was to, first, describe the advantages and disadvantages of gaining soft tissue with a self-inflating soft tissue expander before performing a bone augmentation procedure in implant dentistry in the esthetic zone. Second, we describe how an amalgam tattoo, caused by a previously performed apicoectomy that made the extension of the raised flap to cover the augmented site esthetically undesirable, was removed. Two silicone enveloped Osmed hydrogel self-inflating soft tissue expanders were placed submucosally on the right- and left-hand side of the amalgam tattoo. One of these two perforated the overlaying mucosa after 24 days. Both tissue expanders were removed, the amalgam tattoo was excided, the site augmented, and an implant with a crown and a pontic was placed.

## Introduction

A missing dental unit can be replaced by a (partial) denture, a fixed partial denture (bridge), or a dental implant with a single-unit crown as a superstructure. To be able to place a dental implant, there must be sufficient bone volume. 1 The latter is not always present which in many cases means that a bone augmentation procedure must be performed. 2 3 While performing a bone augmentation procedure, a tension-free closure of the wound is a prerequisite for adequate healing. 4 However, as the inserted bone augmentation material often creates more volume than the available soft tissue flap can cover, an extension of the raised soft tissue flap is required to be able to close the wound in a tension free procedure. The most common procedure then followed to extend the flap is to split the periosteum. However, this procedure does not always provide sufficient soft tissue to allow for a tension-free wound closure. The lack of appropriate soft tissue due to a color or texture mismatch can also make an extension of the raised flap difficult or undesirable. Gaining soft tissue of the appropriate color and texture before augmentation could be a possible solution. 5 6

Soft tissue expansion is a natural phenomenon seen in, for example, pregnancy, obesity, and tumor growth. Soft tissue expansion as a medical application was developed and refined during decades. Neumann introduced it by using a rubber balloon in 1957. 7 Radovan developed it further with intermittent filling injections of saline into the expander in 1984. 8 Austad and Rose described a self-filling tissue expander, using a permeable silicone balloon filled with a saturated NaCl solution in 1982. 9 Wiese started developing self-inflating hydrogel-based tissue expanders in 1983 10 which ultimately resulted in the Osmed hydrogel self-inflating soft tissue expanders (Ilmenau, Germany).

**Fig. 1:**
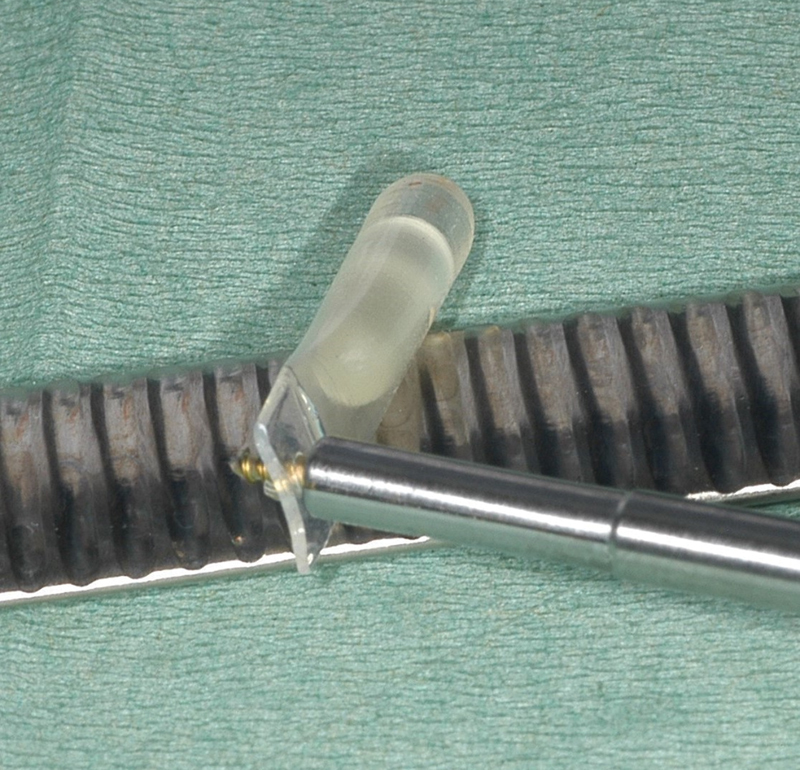
Osmed hydrogel soft tissue expander before insertion in its silicone envelope with a screw through the extension.

The Osmed expander consists of a hydrogel which, after insertion, will increase in volume due to osmosis, making it possible to absorb tissue fluids. Kobus was the first to describe the use of Osmed expanders intraorally in 2007. 11 He used Osmed expanders without a silicone envelope and experienced that the expansion rate was too high. When the hydrogel tissue expander is enveloped in a perforated silicone sheath–like envelope with an extension, this modification reduces the swelling speed ( Fig. 1 ). The extension can be used to secure the expander. The size and number of perforations of the silicone envelope limits the amount of tissue fluids that can pass through to reach the hydrogel and thus the swelling speed of the expander. The size of the envelope determines the maximum expansion volume of the expander that can be achieved. In this case report, we had opted to place enveloped hydrogel expanders because (1) hydrogel expanders when compared with for instance silicone balloon expander have no filling moments, thus avoiding regular office visits; and (2) the dimensions at insertion time are smaller.

The aim of this case report is to describe the advantages and disadvantages of gaining soft tissue with a self-inflating soft tissue expander before performing a bone augmentation procedure in implant dentistry.

## Case Report

To gain soft tissue area and create space under the soft tissue allowing for a bone augmentation procedure, we have been using Osmed (352–3210-S Cylinder 2.1 mL slow, Ilmenau, Germany) hydrogel self-inflating soft tissue expanders. The extension at the end of the silicone envelope was used, to prevent the expander to move during the expansion phase by securing it in the bone at the desired location, with a Straumann cross-head mini screw (Straumann, Basel, Switzerland).

**Fig. 2:**
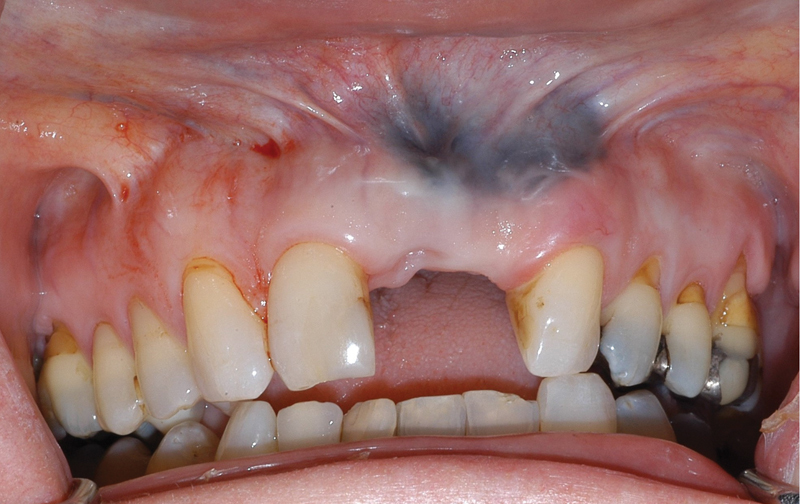
Initial situation showing missing 21 and 22, scar tissue, and an amalgam tattoo.

A patient was referred to our clinic who, after extensive endodontic treatment, apicoectomy, and external root resorption, lost the 21 (a crowned tooth) and the attached pontic 22. Due to financial reasons, she requested if the 21 and 22 could be replaced by only one dental implant and a one-unit pontic. However, due to the lack of sufficient bone volume, a bone augmentation was priorly required if we were to place a dental implant. The mucosa showed an amalgam tattoo with scar tissue as a result of a previously performed apicoectomy ( Fig. 2 ). This made the extension of the raised flap to cover the augmented site esthetically disadvantageous. To overcome this problem, it was discussed with and agreed by the patient, according to the declaration of Helsinki, to excide the amalgam tattoo with the scar tissue after gaining neighboring soft tissue area, using a procedure which involved the use of a soft tissue expander.

**Fig. 3:**
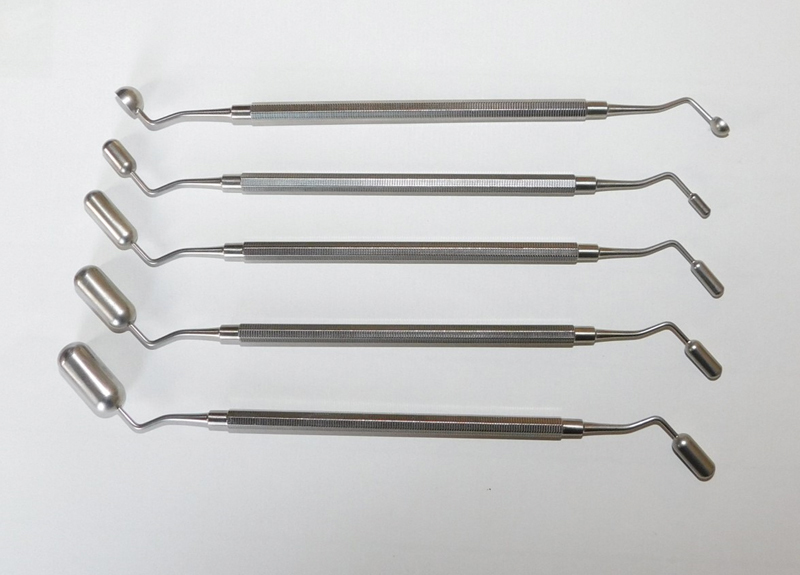
Templates (Osmed) in different dimensions with on the right-hand side the initial size of the expander and on the left-hand side its maximum expanded size.

**Fig. 4:**
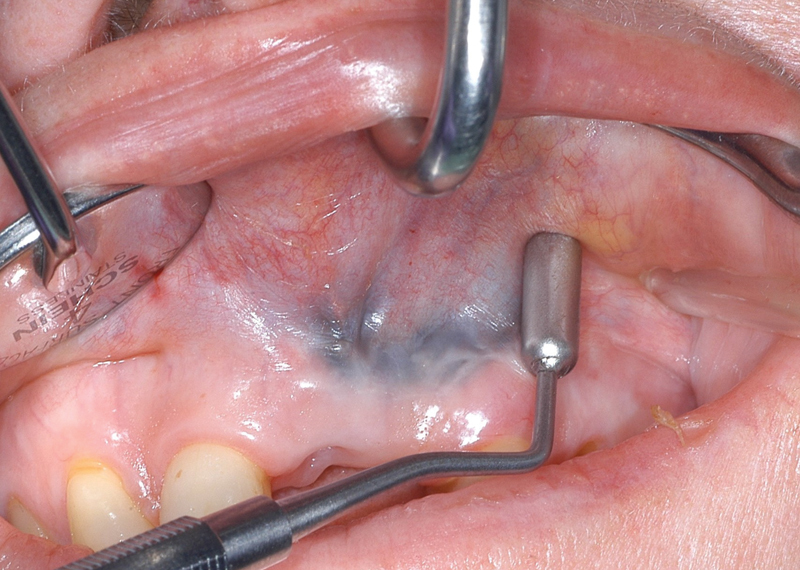
Template left-hand side. It shows the side of the template representing the initial tissue expander size.

**Fig. 5:**
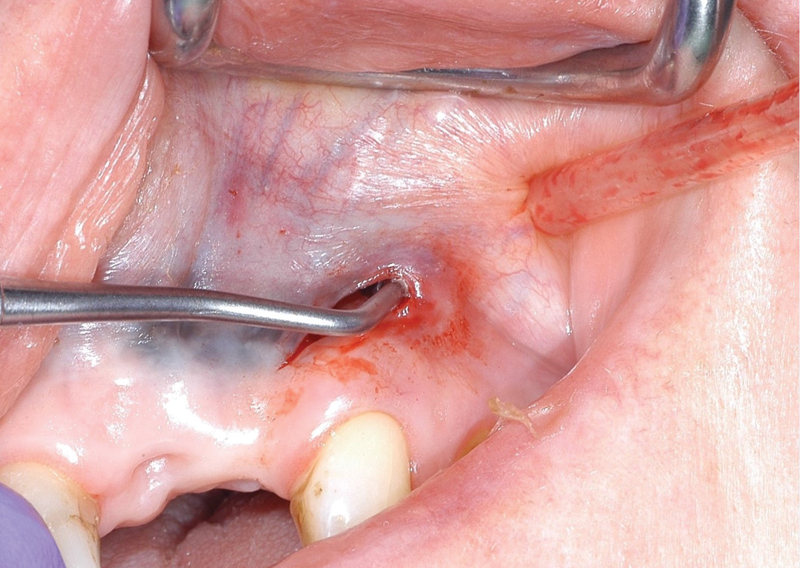
The side of the template representing the initial tissue expander size submucosally in the created tunnel left-hand side.

The tissue expander has to be placed next to the tissue which has to be excided, 6 12 so the gained soft tissue can be used to replace the excided tissue and thus enabling extension of the raised flap. The tissue which has to be excided should not be expanded! The patient is prescribed antibiotics (amoxicillin 500 mg; three tablets/day for 1 week) and an analgetic (ibuprofen 600 mg to be used when pain is experienced). 12 A template (Osmed, Ilmenau, Germany) was used to determine the correct expander size ( Figs. 3 , 4 , 5 ). This template on one side has the dimension of the nonexpanded expander, and on the opposite side, the dimension of the expanded expander ( Fig. 3 ). Before making the incision, care should be taken that the future suture will not extend into the area of the soft tissue that is to be expanded. After the incision was made, a tunnel was prepared with a blunt instrument by raising overlaying mucosa from the bone. To check if the size of the created tunnel was sufficient to fit the expander, the side of the template representing the initial tissue expander size was inserted into the created submucosal space ( Fig. 5 ). A small incision, perpendicular to the first one, was made to be able to secure the screw in the extension. The hydrogel expander (Osmed 352–3210-S Cylinder 2.1 mL slow, Ilmenau, Germany) was secured with one Straumann cross-head mini screw (Straumann, Basel, Switzerland) through the extension of the silicone envelope to overcome possible dislocation which can occur due to the expansion. The wound was closed with a saliva-tight suture.

**Fig. 6:**
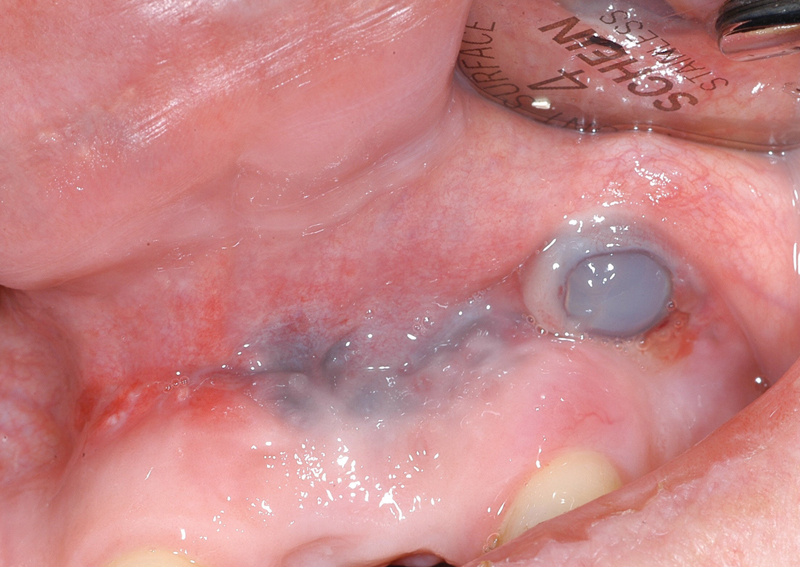
Perforation left-hand side after 24 days, a surplus of soft tissue cranial of the incision.

Both Osmed hydrogel tissue expanders were submucosally placed at *t* = 0. The left-hand-side tissue expander perforated its overlaying mucosa after 22 days and the mucosa defect increased ( Fig. 6 ). Therefore, both tissue expanders were removed after 24 days together with their securing screws. The amalgam tattoo was excided, the bone site augmented with Bio-oss (Geistlich Pharma AG, Bahnhofstrasse 40, 6110 Wolhusen, Switzerland) and covered with Bio-guide (Geistlich Pharma AG, Bahnhofstrasse 40, 6110 Wolhusen, Switzerland). As we had gained sufficient soft tissue, we could close the wound in a tension-free manner. We did not notice any excessive bone loss due to the possible pressure of the soft tissue expander on the bone during the soft tissue expansion procedure. A Straumann (Straumann Holding AG, Peter Merian-Weg 12, 4002 Basel, Switzerland) implant was placed 6 months later.

**Fig. 7:**
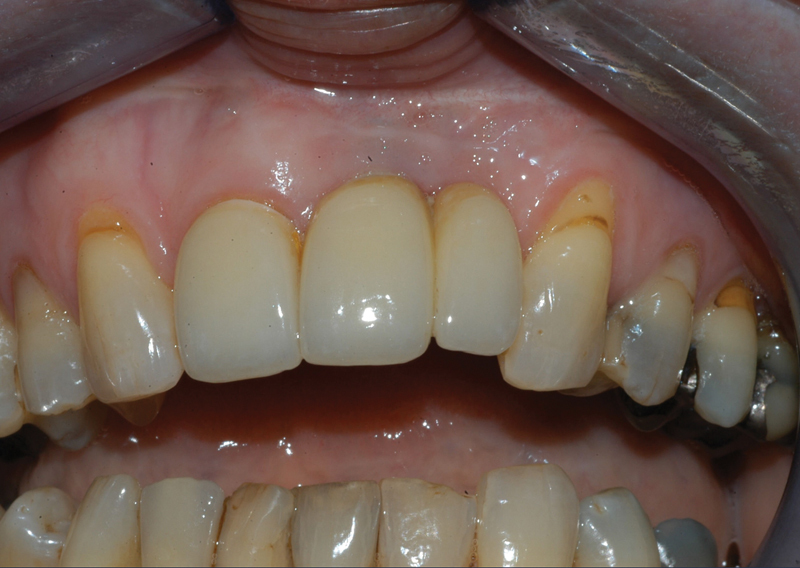
Intraoral view after 8 years.

After treatment, the patient visited the dental office for her biannual check-ups on a regular basis. After 8 years, the situation is still acceptable for our patient ( Fig. 7 ), even though it is noticeable that the contour of the buccal implant mucosa is not attractive and the papilla is missing. One can also see that color of the mucosa at the location where the amalgam tattoo was excided is a little different. That being it, the patient is satisfied that the previous amalgam tattoo is no longer visible.

## Discussion

In reconstructive plastic surgery, soft tissue expansion is a common surgical procedure which is often performed to develop donor tissue that matches the surrounding tissue in color, texture, and thickness. 7 8 13 The model that is most often used is as silicone balloon with a remote injection port, which is gradually filled with saline, thus increasing in volume. However, the silicone balloon model requires more space when placed compared with the hydrogel expander. In addition, the tube from the external filling port also needs space or is placed in a submucosal tunnel that ends extraorally. When using an intraoral silicone balloon expander without an external filling port, regular visits to the dental office are necessary, as it has to be filled to continue its inflation during several weeks.

The hydrogel-based tissue expanders consist of osmotically active cross-linked copolymers based on methyl-methacrylate (MMA) and N-vinylpyrrolidone. Because they swell rapidly as soon as they take up fluids, which can result in a volume increase of over 500% within 24 to 48 hours, they can perforate the overlaying tissue. 11 14 This depends on the available tissue fluid, pressure within the tissue space, experience of the surgeon, size of the chosen expander, as well as the tension of the overlying soft tissue. If a tissue expander is chosen that is too large in size, this will result in a soft tissue perforation, as well as a too rapid expansion. To minimize the risk of soft tissue perforation and to reduce expander inflation speed, they are enveloped in a silicone hull (envelope) which regulates the amount of fluid being absorbed by the expander. The difference between the behavior of enveloped and nonenveloped tissue expanders becomes visible after a few days when both have reached 20% of their maximum reachable volume. After 7 days, a nonenveloped one reaches 48%, whereas an enveloped one reaches 30%, 60% and 40% after 16 days, respectively. 15 The slower expansion speed will give its overlaying tissues more time to adapt. The maximum reachable volume of the tissue expander can be determined by the size of the envelope. Once the osmotic tissue expander has been placed, its expansion is not influenceable and the enveloped one reaches its maximum size after approximately 40 days, whereas the nonenveloped reaches this after approximately 16 days. 15

If a treatment device with unknown characteristics to the surgeon is used, the patient should be seen very often and the device removed as soon as there are signs of malfunction. For example, one could think of severe pain, inflammation, ischemia, or fistula. 16 In this case, we removed the tissue expanders before they could reach their maximum expansion size. In this particular case, the tissue expander on left-hand side was placed more to the mucogingival margin compared with the expander right-hand side ( Fig. 6 ). The perforation of the tissue expander on left-hand side was just above the mucogingival margin. Here, the gingiva is more firmly attached to the bone compared with the mucosal tissues and was not separated from the bone before the expander was inserted. This means that as the expander expands, it is not to be expected that the gingiva will expand. At expanding, it will follow the path of least resistance. If the expansion speed of the expander goes above the stretch characteristics of its overlaying tissue, the expander will perforate the tissue, as unfortunately in this case, it happened on the left-hand side. To overcome this undesired event, a tissue expander with a lower expansion “speed” should have been used and a larger soft tissue area should be separated from the bone. A smaller tissue expander could have been inserted; however, one must also be sure that it is large enough to obtain the desired amount of tissue.

Soft tissue expansion before performing bone augmentation procedures in the oral cavity leads to extra operating and healing time, as well as extra costs. 6 There is a need of more sophisticated, small, intraorally easy to use, tissue expanders. Articles have been published on a novel reshapeable hydrogel tissue expander, 17 18 19 but this expander is not available on the market at this time. We would like to encourage, like other authors also have done, 20 additional research in this field.

## Conclusion

With this case report, we want to once again draw attention to the promising possible intraoral use of tissue expansion, but also next to its advantages also point out some difficulties. With the appropriate use in the correct case, there is certainly an indication application for future intraoral soft tissue expansion. Important considerations in future applications are the swelling speed, the location of the tissue expander, and surgical techniques applied among others.
